# Novel technique for biliary reconstruction using an isolated gastric tube with a vascularized pedicle: a live animal experimental study and the first clinical case

**DOI:** 10.1186/1750-1164-5-8

**Published:** 2011-10-10

**Authors:** Ashraf A Helmy, Mostafa A Hamad, Ahmed M Aly, Tahra Sherif, Mostafa Hashem, Dalia AH El-Sers, Mohammad Semieka

**Affiliations:** 1 Department of General Surgery, Faculty of Medicine, Assiut University, Assiut, Egypt; 2 Department of Clinical Pathology, Faculty of Medicine, Assiut University, Assiut, Egypt; 3 Department of Radiology, Faculty of Medicine, Assiut University, Assiut, Egypt; 4 Department of Pathology, Faculty of Medicine, Assiut University, Assiut, Egypt; 5 Department of Veterinary Surgery, Faculty of Veterinary Medicine, Assiut University, Assiut, Egypt

## Abstract

**Background:**

Biliary tract reconstruction continues to be a challenging surgical problem. Multiple experimental attempts have been reported to reconstruct biliary defects with different materials and variable outcome. Our aim was to evaluate a new method for biliary reconstruction using an isolated pedicled gastric tube in a live animal trial and also to present the first clinical case.

**Methods:**

Seven mongrel dogs underwent biliary reconstruction using gastric tube harvested, completely separated from the greater curvature, and based on a vascularized pedicle with the right gastroepiploic vessels. The tube was interposed between the common bile duct (CBD) and the duodenum. Postoperative mortality, morbidity, liver functions, gross and microscopic histological picture were assessed. The first clinical case was also presented where, in a patient with post-cholecystectomy biliary injury, an isolated pedicled gastric tube was interposed between the proximal and distal ends of the CBD.

**Results:**

One dog did not recover from anesthesia and another one died postoperatively from septic peritonitis. Five dogs survived the procedure and showed uneventful course and no cholestasis. The mean anastomotic circumference was 4.8 mm (range 4-6) for CBD anastomosis and 6.2 mm (range 5-7) for duodenal anastomosis. Histologically, anastomotic sites showed good evidence of healing. In the first clinical case, the patient showed clinical and biochemical improvement. Endoscopic retrograde cholangiography was feasible and assured patent biliary anastomoses.

**Conclusion:**

In mongrel dogs, biliary reconstruction using pedicled gastric tube interposition between CBD and duodenum is feasible with satisfactory clinical results, anastomotic circumference and histological evidence of healing. The technique is also feasible in human and seems to be promising.

## Introduction

Biliary injuries as a result of cholecystectomy and other biliary operations are complex and serious. The frequency has not diminished and probably will not. Even as the knowledge advances, lesions continue to occur and remain as problems for the surgeon [1].

In many instances, when the duct has not lost continuity, interventional radiological and/or endoscopic approach can be used with good results. Nevertheless, complete section of the duct requires surgical reconstruction [2]. Currently, unless the lesion is located in the distal common bile duct (CBD), the reconstruction of choice is mucosa to- mucosa Roux-en-Y loop hepaticojejunostomy [3].

Additionally, regarding iatrogenic biliary strictures, Roux-en-Y hepaticojejunostomy is the standard procedure of choice [4]. Other significantly less common indications for hepaticojejunostomy include biliary fibrosis produced by chronic pancreatitis, penetrating trauma of the porta hepatis, previous bilioenteric operation with subsequent stricture formation, choledochal cyst resections and other causes of iatrogenic biliary trauma such as gastric, pancreatic or hepatic resections, portal decompression procedures and liver transplantation. Malignant conditions such as cholangiocarcinoma and gall bladder carcinoma infiltrating CBD or hepatic ducts may also indicate hepaticojejunostomy as a final step of the resective procedure or as a palliative attempt to relieve jaundice in instances of unresectability [5]. However, 7 to 38% of patients with hepaticojejunostomy have been reported to develop biliary complications as cholangitis, recurrent anastomotic strictures and hepatodocholithiasis, which, if left untreated, may lead to liver cirrhosis and portal hypertension. Although some of these long-term complications may be treated by means of interventional radiology without the need for re-operation, transhepatic manipulations can be difficult, represent temporary solution and are prone to complications [6].

In this study, we are introducing a new technique for reconstruction of the biliary tract using isolated gastric tube with vascularized pedicle as a potential alternative for hepaticojejunostomy. This technique is tested in a live animal experimental study. Additionally, we are presenting the fist clinical case using this biliary reconstruction technique.

## Materials and methods

The detailed study protocol was approved by the *Research Ethical Committee *of Assiut Faculty of Medicine and the trial was, afterwards, performed in the period from November 2009 till August 2010.

Seven mongrel dogs of both sexes weighing 16 to 22 kg were anaesthetized, after 8 hours fast, using intravenous pentobarbital (30 mg/kg). They were intubated with cuffed endotracheal tube and allowed to ventilate using 2% halothane inhalation vaporized with 100% oxygen. At the time of induction of anesthesia, intravenous amoxicillin (5 mg/kg) was given.

Midline abdominal incision was used to expose the gall bladder and the CBD which is transected immediately distal to the confluence of right hepatic duct with extension of the incision in the right hepatic duct to produce a wider stoma.

**Figure 1:**
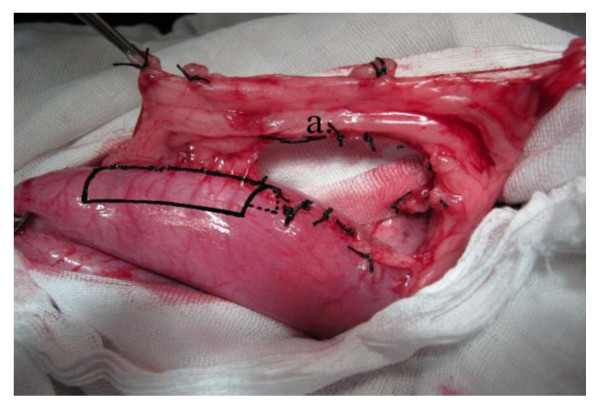
**Operative photo**: Shows mobilization of the right. gastroepiploic vessels from the greater curvature of the stomach. a) Right gastroepiploic vessels.

**Figure 2:**
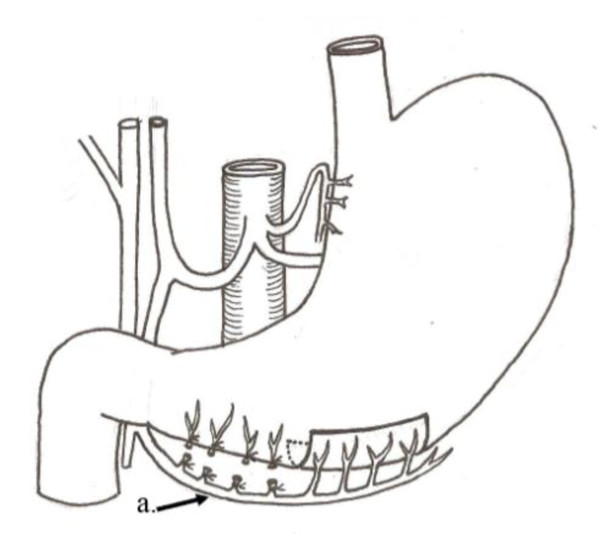
**Operative diagram**: Illustrates figure 1. a) Right gastroepiploic vessels.

**Figure 3:**
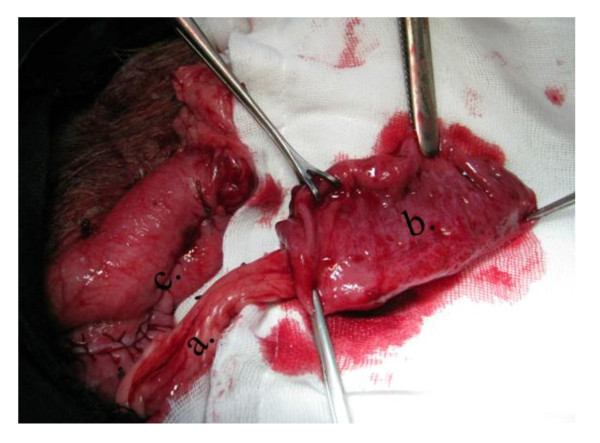
**Operative photo**: Showing a pedicled piece of the stomach wall based on the right gastroepiploic vessels. a) Right gastroepiploic vessels. b) Piece from the middle 1/3 of the greater curvature of the stomach. c). Closure of defect at the greater curvature of the stomach.

**Figure 4:**
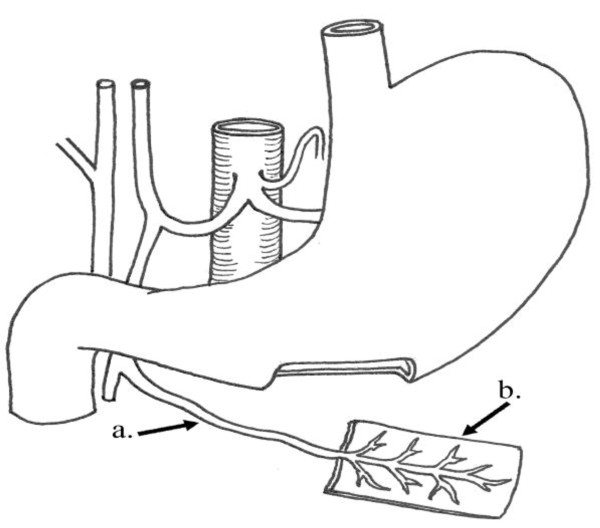
**Operative diagram**: Illustrates figure 3. a) Right gastroepiploic vessels. b) Piece from the middle 1/3 of the greater curvature of the stomach.

**Figure 5:**
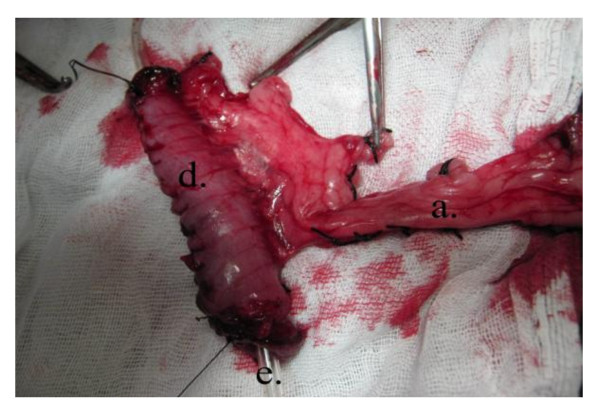
**Operative photo**: Showing fashioning of gastric tube over a catheter. a) Right gastroepiploic vessels. d). Piece from the stomach wall fashioned as a tube. e) catheter.

**Figure 6:**
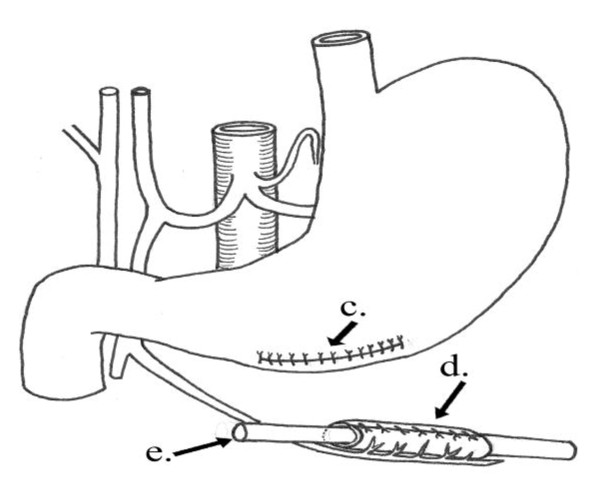
**Operative diagram**: Illustrates figure 5. c) Closure of defect at the greater curvature of the stomach. d) Piece from the stomach wall fashioned as a tube. e) Catheter.

**Figure 7:**
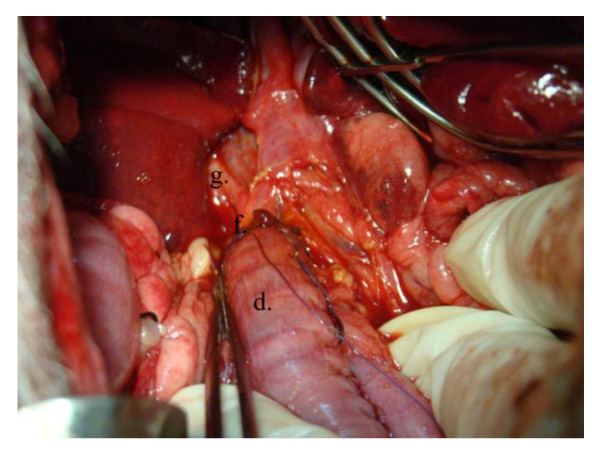
**Operative photo**: Showing the anastomosis between the distal end of CBD to the fashioned gastric tube. d) The pedicled gastric tube. f) Anastomosis of the gastric tube to the CBD at the site of confluence of the right hepatic duct. g) Right hepatic duct.

**Figure 8:**
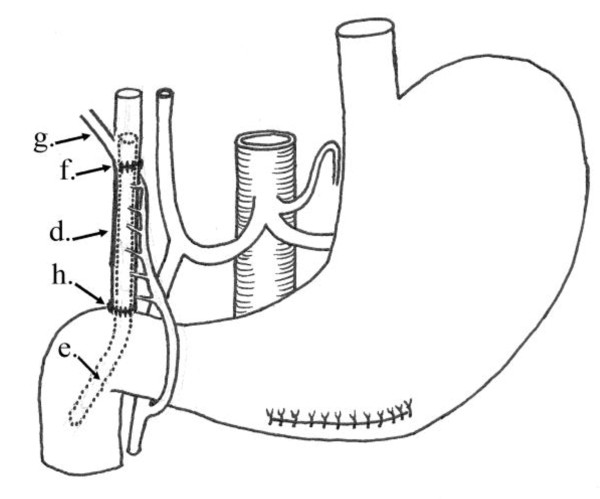
**Operative diagram**: Illustrates anastomosing the newly fashioned gastric tube to both the distal end of CBD and the duodenum. d) The pedicled gastric tube. e) Silicon catheter. f) Anastomosis of the gastric tube to the CBD at the site of confluence of the right hepatic duct. g) Right hepatic duct. h) Anastomosis of the gastric tube to the duodenum.

**Figure 9:**
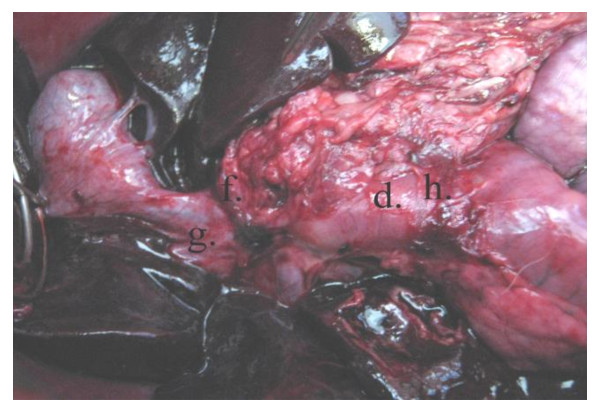
**Postmortem specimen**: Showing anastomosis of the newly fashioned gastric tube to both the distal end of CBD and the duodenum. d) The pedicled gastric tube. f) Anastomosis of the gastric tube to the CBD at the site of confluence of the right hepatic duct. g) Right hepatic duct. h) Anastomosis of the gastric tube to the duodenum.

A piece from the middle 1/3 of the greater curvature of the stomach, 10-15 cm from pylorus and 5 × 3 cm in diameter, was completely separated from the stomach with its blood supply based upon right gastroepiploic vessels which were mobilized by meticulous dissection (Figure 1, 2, 3 and 4). The resulted defect in the stomach was closed in two layers. The piece of the stomach wall was fashioned as a tube over a silicon catheter of 4 mm diameter which later acted as an anastomotic stent (Figure 5 and 6). The distal part of the CBD was anastomosed to the newly fashioned tube which in turn anastomosed to the duodenum 2-3 cm distal to the pylorus (Figure 7, 8 and 9). Both anastomoses were mucosa to mucosa using 5/0 Vicryl (Ethicon inc., Johnson & Johnson company) interrupted sutures. The posterior row of sutures was placed followed by the anterior row. The stent was fixed by a stitch of the same suture type to the CBD wall to prevent slipping and its distal tip was placed in the duodenum.

After thorough haemostasis, the abdominal cavity was washed with saline and closed with intraperitoneal suction drain in position. Prior to extubation, intramuscular tramadol HCl 6 mg/kg was given and thereafter daily for three days for postoperative analgesia.

Postoperatively, all dogs were fed with standard dog chow and allowed free access to water and free movement in their cages. Intravenous amoxicillin 5 mg/kg were given every 8 hours for 10 days.

The animals were followed up for any biliary complications in the form of obstructive jaundice, cholangitis or biliary leakage.

Blood samples were obtained from the jugular vein of the canine both at the time of surgery and six weeks later at the time of euthanasia. Samples were allowed to clot for 30 minutes prior to centrifugation at 1500 × g for seven minutes and subsequently stored in plastic Eppendorf tubes at -20°C for no longer than one month until analysis. Liver functions tests were performed including serum total bilirubin, alkaline phosohatase (ALP), alanine aminotransferase (ALT) and aspartate aminotransferase (AST). These tests were performed on "Hitachi 911 automatic analyzer" (Boehringer-Mannheim). The normal range for dogs are (0.1-0.6 mg/dL) for bilirubin, (10.6-101 U/L) for ALP, (8.2-57 U/L) for ALT and (8.9-49 U/L) for AST [7].

**Figure 10:**
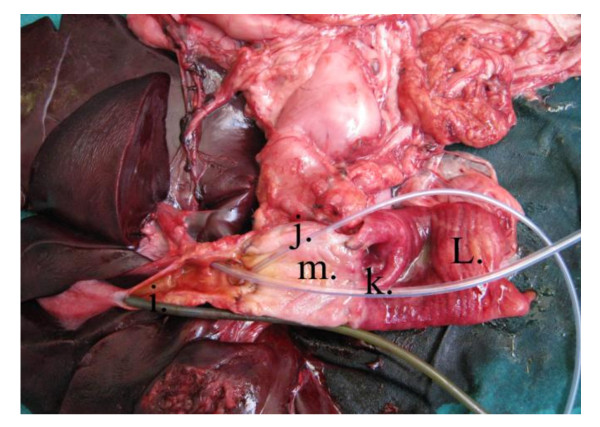
**Postmortem specimen**: Showing the interior of the pedicled gastric tube, the biliary tract and the duodenum. i) Tube into gall bladder lumen. j). Tube in the right hepatic duct. k) Tube in the left hepatic duct. l) Interior of the duodenum. m) Interior of the pedicled gastric tube.

**Figure 11:**
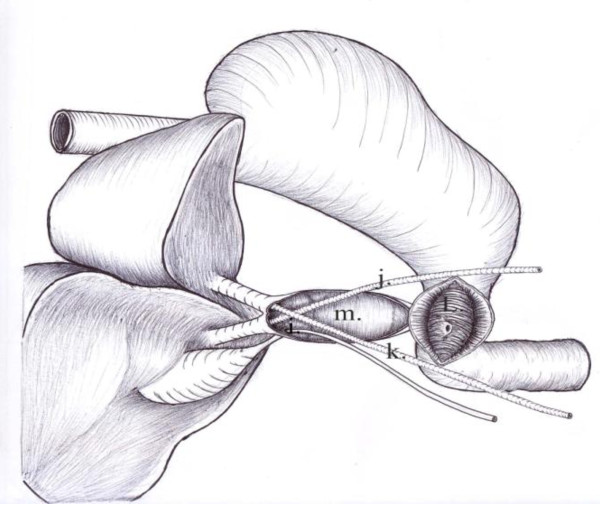
**Postmortem diagram**: Illustrates figure 10. i) Tube into gall bladder lumen. j). Tube in the right hepatic duct. k) Tube in the left hepatic duct. l) Interior of the duodenum. m) Interior of the pedicled gastric tube.

**Figure 12:**
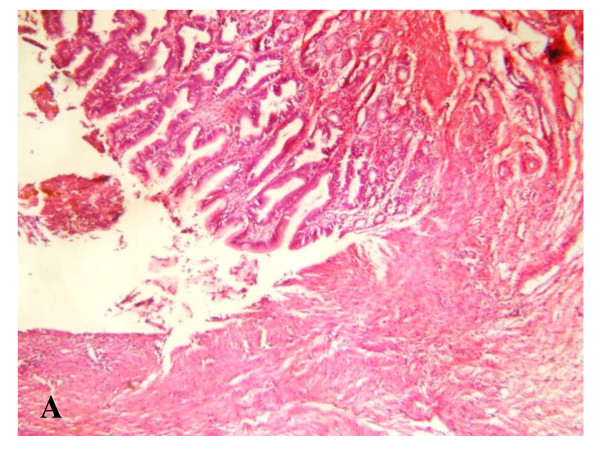
**Microscopic examination**: Showing the anastomotic line between the pedicled gastric tube and common hepatic duct, ×4.

**Figure 13:**
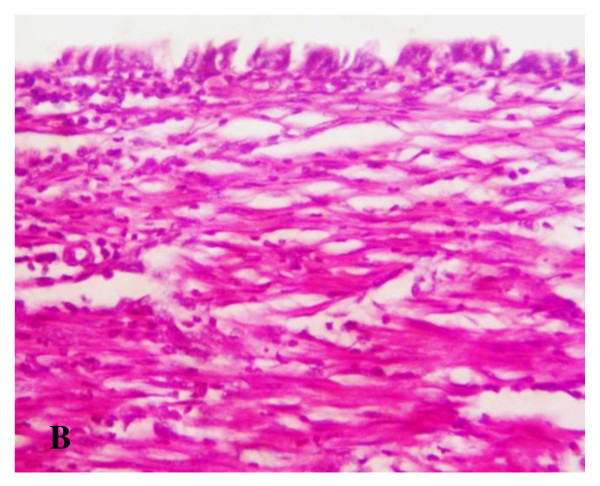
**Microscopic examination**: Showing the common hepatic duct showing regeneration of the epithelium. The submucosa reveals the presence of proliferating fibroblasts with collagen deposition and infiltration with inflammatory cells, ×20.

**Figure 14:**
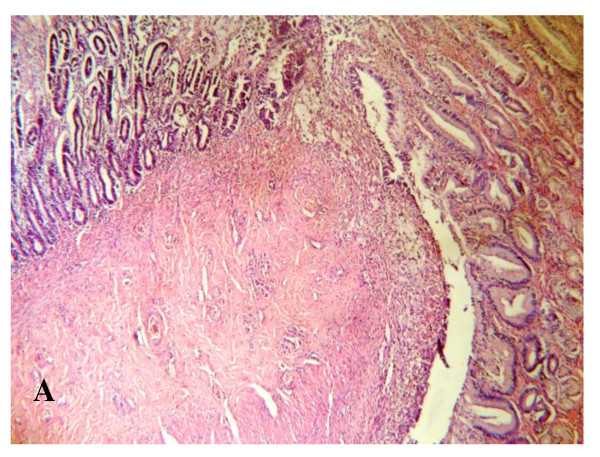
**Microscopic examination**: Showing the anastomotic site between the pedicled gastric tube and duodenum, ×10.

**Figure 15:**
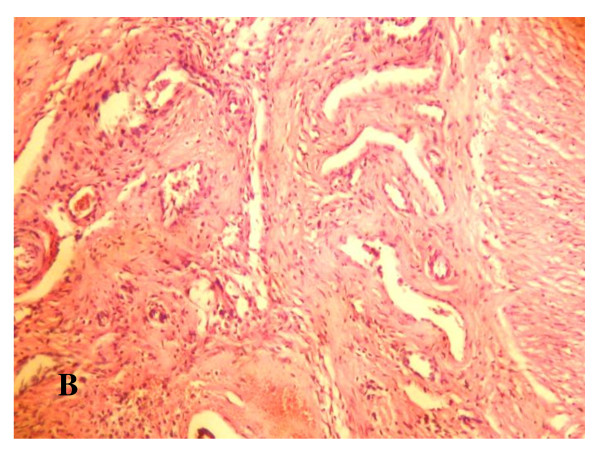
**Microscopic examination**: Showing the submucosa at the junction between pedicled gastric tube and duodenum showing proliferating blood capillaries, proliferating fibroblasts with collagen deposition, and infiltration with inflammatory cells ×20.

Six weeks after the procedure, the abdomen was explored again under general anesthesia for any biliary leak, intraabdominal collection, disrupted anastomosis or anastomotic stricture. An operative specimen was obtained including the liver, extra hepatic biliary channels, gastric tube, duodenum and stomach before sacrifice of the animal (Figure 9). Grossly, we measured the circumference of the anastomoses of the gastric tube with both CBD and duodenum (Figure 10 and 11). Thereafter, the specimens had been fixed in 10% formalin for 24 hours before they were trimmed. Sections were routinely processed. Five micron-thick sections were cut from paraffin blocks, and stained with hematoxylin and eosin stain for histological examination (Figure 12, 13, 14 and 15).

First clinical case: in December 2010, a 45 year female presented to the authors with external biliary leak two weeks after open cholecystectomy. The patient had a past history of exploration for rupture mesenteric cyst with resection anastomosis of part of the small intestine five years before. Abdominal ultrasonography showed moderate right sub hepatic biliary collection. Serum bilirubin, alkaline phosphatase and liver aminotransferases were elevated. Endoscopic retrograde cholangiopancreatography showed complete ligation of the distal end of the CBD. After obtaining an informed consent from the patient explaining all the possibilities of surgical procedures including the isolated pedicled gastric tube technique, she was explored through generous right subcostal incision with upward midline extension. Extensive adhesions was taken down to reach the proximal CBD about one cm from the confluence of the right and left hepatic ducts. The distal CBD end was dissected and the ligature removed with confirmation of the patency of the sphincter of Oddi. The gap between the proximal and distal CBD ends was about three cm. Construction of a Roux-en-Y jejunal limp seemed very difficult due extensive adhesions from the past operative procedure for intestinal resection. The high position of the proximal CBD end made hepaticoduodenostomy inappropriate. End to end choledochocholedochostomy was not an option due to the high rate of future stricture and the long gap between the two CBD ends. Consequently, the surgeons preferred to use the new technique of isolated pedicled gastric tube interposition. Because the distal CBD was intact, we decided to interpose the gastric tube between both ends of the CBD to keep the sphincter of Oddi physiologically intact. A four mm stent was left in position from the proximal end of the CBD through the gastric tube to the duodenum and fixed to the CBD wall with fine 5/0 Vicryl suture. The patient was followed up clinically and by liver function tests and endoscopic retrograde cholangiopancreatography (ERCP). Written informed consent was obtained from the patient for publication of her data and any accompanying images.

## Results

Two dogs died; one intraoperatively from anesthetic complication and the other dog died three days later from septic peritonitis with neither evidence of biliary leak nor cholangitis was found in postmortem examination. The cause of septic peritonitis was obscure although minor iatrogenic bowel perforation which had sealed spontaneously later on was suspected. Both dogs were excluded from the study. The other five dogs survived the first operation. The operative time ranged from 130 to 185 minutes with a mean of 156 minutes.

One dog experienced mild biliary leak which, thereafter, stopped spontaneously on the fifth postoperative day. There were no clinical evidence of other biliary complications as obstructive jaundice and cholangitis. Preoperatively, the liver functions in the form of bilirubin, ALP, ALT and AST were normal. Six weeks after the procedure, the liver functions remained within normal range for dogs (table 1).

**Table 1:** Liver function tests

Animal	Bil (μmol/L)			ALP (u/L)			ALT (u/L)			AST (u/L)		
	
	N	Pre	Post	N	Pre	Post	N	Pre	Post	N	Pre	Post
**No. 1**	0.9-10.6	3.2	4.7	10.6-101	25.9	40.3	8.2-57	47.2	55.1	8.9-49	15.4	40.6
**No. 2**		5.6	7.3		44.7	37.5		16.5	31.1		32.5	49.2
**No. 3**		1.9	4.4		70.6	82.0		40.5	13.3		38.0	21.7
**No. 4**		4.6	3.5		16.3	33.6		38.6	48.7		12.3	38.1
**No. 5**		6.0	2.7		63.6	28.8		22.6	41.4		28.8	23.9

Showing values preoperatively and six weeks after the procedure in relation to the normal range.

N: normal values, Bil: bilirubin, ALP: alkaline phosphatase, ALT: alanine aminotransferase, AST: aspartate aminotransferase, N: normal, Pre: preoperative, Post: postoperative

Postmortem gross examination of the specimens revealed patent lumen of the pedicled gastric tubes (Figure 10 and 11). The mean anastomotic circumference was 4.8 mm with a range from 4 to 6 mm for the CBD anastomosis and a mean of 6.2 mm for the duodenal anastomosis with a range from 5 to 7 mm. Microscopic examination of the gastric tube and CBD showed evidence of healing in the form of angiogenesis, proliferating fibroblasts and collagen depositions in the submucosa at the anastomotic sites. The covering epithelium showed regenerative changes with no evidence of cholangitis (Figure 12, 13, 14 and 15). Microscopic examination of the liver revealed no evidence of cholangiohepatitis.

**Figure 16:**
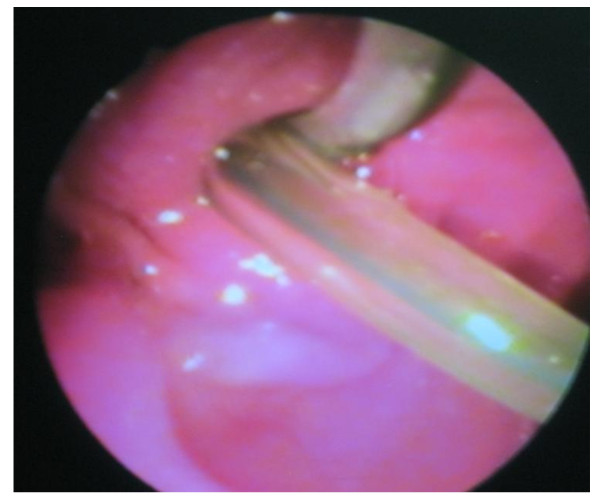
**Postoperative endoscopic picture**: Showing the duodenal papilla with both the stent and the balloon catheter passing through the papillary orifice.

**Figure 17:**
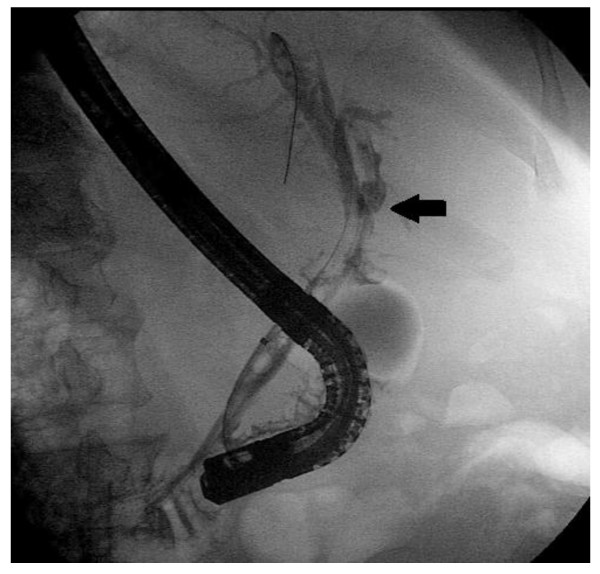
**Postoperative ERCP**: Showing the gastric tube anastomosed to the proximal CBD end (arrow). The gastric tube anastomosis with the distal end is masked by the endoscope shaft. The inflated balloon catheter accompanied by the stent is seen within the reconstructed biliary tract.

Results of the first clinical case: the patient showed clinical improvement within one week and discharged from the hospital within ten days. Liver functions showed improvement within two weeks. Four weeks after the operation, standard ERCP showed patent anastomoses between the gastric tube from one side and the proximal and distal CBD ends from the other side (Figure 16 and 17). The patient had been followed up for five months with no reported biliary complications.

## Discussion

Multiple experimental attempts have been reported to replace biliary defects with a variety of materials. However, many series failed to support the usefulness of these materials when placed in the extra hepatic canine biliary tract. Mandelowits and Beal [8] used expanded polytetrafluoroethylene in reconstruction of the canine biliary system of eleven mongrel dogs. They found that the Teflon graft was inadequate due to rejection and fibrosis resulting in partial or complete obstruction of the duct. When they attempted to line the graft material by autogenous vein, the results were also unsatisfactory. Autolysis of the vein, rejection of Teflon into the lumen of the common bile duct and circumferential scarring were the primary responses at 30 to 40 days.

Other authors tried to use autologus veins for reconstruction of the bile ducts with conflicting results. Amiranashvili and his group [9] used auto-venous transplants to perform hepaticocholedochal reconstruction in 10 mongrel dogs. They found that auto-venous transplant was subjected to incrustation by bile salt and later on it became necrotic and concluded that this technique could not be used in biliary reconstruction. On the contrary, Capitanich et al. [10] found that the use of an autologous vein graft with a supporting stent proves to be a feasible and alternative procedure for bile duct reconstruction in rats. Moreover, Palmes et al. [11] in a pig model reported that neo-bile duct was created using a segment of the external jugular vein which was endoluminally stented by a biodegradable poly-lactate-acid stent. The neo-bile duct morphologically resembles native bile duct with no reported cholestasis.

Rosen et al. [12] have, experimentally, described a method of biliary tract regeneration using a porcine small intestinal submucosa, another substitute material that leaves no foreign matter in the body. Yet small intestinal submucosa also has drawbacks, because it leaves the patient susceptible to zoonotic infection and may induce cicatricial contractions [13].

On the other hand, Aikawa et al [1] implanted bioabsorbable polymer patch to a bile duct defect. They found maintained patency which led to duct dilation. It served as a scaffold for the regeneration of tissue similar to the native duct tissue while allowing the bile to drain normally without leaving traces of foreign matter in the body.

In this experimental study, we have assessed a new method of biliary reconstruction using interposition of an isolated gastric tube with vascularized pedicle and also presented the first clinical case using this technique. To our knowledge, this technique is a novel one that is introduced for the first time. Our study showed satisfactory results regarding biliary complications, liver functions and anastomotic circumference. We think that this method could represent a potential new way for biliary reconstruction. The isolated pedicled gastric tube interposition might be advantageous to hepaticojejunostomy because it seems to be more physiologic operation keeping the pathway of bile into the duodenum especially if the gastric tube is interposed between the proximal and distal CBD ends keeping the sphincter of Oddi functioning. On the contrary, bypassing the bile away from the duodenum, Roux-en-Y hepaticojejunostomy is proved to be associated with significant incidence of postoperative peptic ulceration [14]. Additionally, our method, as proved by the clinical case presentation, could easily offer the ability for diagnostic and therapeutic ERCP, a technique that proved to be extremely difficult after hepaticojejunostomy [15]. Moreover the satisfactory anastomotic circumferences in our study could be attributed to the adequate blood supply of the stomach tube provided that the feeding vessels are well preserved. This could be translated into less stricture rates in future clinical assessment if compared to the still problematic rate with hepaticojejunostomy [16]. Other potential advantages of pedicled gastric tube are the light weight of the tube with minimal traction on the anastomosis and also the irrelevance of the presence of short mesentery to the performance of this technique unlike hepaticojejunostomy.

Heimlich and Giltlitz reported biliary reconstruction using pedicled flap of the stomach based on the greater curvature with its end anastomosed to the proximal CBD [17]. The tube is not isolated from the stomach. They encountered high percentage of cholangitis (5 out of 7 dogs) which could be attributed to the high intragastric pressure which may result into ascending reflux cholangitis with cholecystitis if the gall bladder was not removed or to strictured biliary anastomosis manifested with the picture of cholangitis. Our technique differs as the gastric tube is completely isolated from the stomach keeping only the vascular pedicle. The distal anastomosis could be with the distal CBD, as was the case in the experimental trial, or with the duodenum as in the clinical case. Consequently, our technique would not lead to biliary gastritis, as would the case of with Heimlich and Giltlitz technique, because the bile passes to the duodenum either directly or via the distal CBD. Additionally, we were not confronted with evident cholangitis although a longer follow up was needed. The absence of cholangitis in our study could be due to the preservation of the function of the CBD sphincter in the experimental trial and to the anastomosis with the duodenum, which has relatively lower intraluminal pressure than the stomach, in the clinical case. Also we did not have cholangitis due to anastomotic stricture during the study period.

Other studies have reported isolated jejunal loop interposition between the biliary tract and the duodenum as a method of biliary reconstruction whether experimentally [18] or clinically [19,20]. They found that short jejunal loop is associated with higher rate of cholangitis caused by refluxed duodenal contents into the biliary channels but endoscopic biliary access is feasible. On the other hand, long jejunal loop more than 40 cm is associated with less cholangitis but proved very difficult for endoscopic biliary access. The issue of reflux cholangitis with pedicled gastric tube biliary reconstruction awaits further assessment experimentally and clinically. However, the small diameter of the fashioned gastric tube could, theoretically, interfere with reflux of the duodenal contents into the bile duct. On the other hand, endoscopic biliary access was proved feasible with this technique as shown in the clinical case presentation. Furthermore, the length of the gastric tube could be adjusted to exactly bridge the biliary defect; a point that might be of help to the biliary endoscopic techniques whether diagnostic or therapeutic.

In the case of bilioenteric anastomosis, some authors are relating the relapsing cholangitis, caused by reflux of intestinal fluid into the biliary channels, to the increased possibility of cholangiocarcinoma in those patients [21]. In this context, further work needs to be done to explore the feasibility of using pedicled gastric tube as an interposed segment between the proximal and distal end of the CBD leaving the duodenal papilla intact to prevent recurrent ascending cholangitis with its complications. This was the reconstruction pattern used in the first clinical case reported in this study. However, the assessment of theoretical risk of malignant metaplasia of the gastric tube mucosa, due to chronic exposure to bile, needs prolonged follow up of large number of cases.

## Conclusion

We conclude that, in mongrel dogs, reconstruction of the biliary tract using interposition of an isolated gastric tube with a vascularized pedicle between CBD and duodenum is feasible and produces satisfactory results regarding biliary complications, anastomotic circumference and histological evidence of healing. This technique is also feasible in human with potential success and possible follow up by standard ERCP. Additional studies are still needed to further evaluate this technique in the clinical setting.

## Competing interests

The authors declare that they have no competing interests.

## Authors' contributions

AAH conceived the design and participated in the operations. MAH participated in conception, designed the study, and drafted the manuscript. AMA participated in the operations and revision of the manuscript. TS performed the biochemical assays and revised the manuscript. MH performed the radiological assessment of the case and participated in the draft. DAHES performed the histological assessment and revised the manuscript. MS participated in the operations and acquisition of data. All authors read and approved the final manuscript.
